# Asystolie au cours d’une chirurgie pour tumeur de l’intestin grêle: anaphylaxie ou crise carcinoïde

**DOI:** 10.11604/pamj.2018.30.92.14877

**Published:** 2018-05-31

**Authors:** Ismail Aissa, Aziz Benakrout, Mohamed Meziane, Mustapha Bensghir, Salim Jaafar Lalaoui

**Affiliations:** 1 Pôle d’Anesthésie-Réanimation, Hôpital Militaire d’Instruction Mohamed V, Faculté de Médecine et de Pharmacie, Université Mohamed V, Rabat, Maroc

**Keywords:** Asystolie, tumeur de l´intestin grêle, anaphylaxie, crise carcinoïde, Asystole, small intestine tumor, anaphylaxis, carcinoid crisis

## Abstract

L'arrêt cardiaque au bloc opératoire est un événement redoutable, ces étiologies sont multiples. Nous rapportons le cas d'une patiente de 53 ans sans antécédents pathologiques particuliers, programmée pour cure chirurgicale d'une tumeur de l'intestin grêle. 20 minutes après l'induction anesthésique la patiente a présenté une asystolie rapidement réversible après les mesures de réanimation, l'association d'un rush cutané du visage et du thorax a fait suspecter une réaction anaphylactoïde tardive. L'amélioration rapide de la patiente a permis l'autorisation d'une reprise de l'intervention. Le début de la manipulation tumorale a occasionné une deuxième bradycardie extrême rapidement résolutive après 0,5 mg d'atropine, une réapparition du rush cutané au niveau du visage et du thorax a été observée, cette deuxième complication a rapidement fait suspecter une crise carcinoïde conduisant à l'administration du sandostatine. Aucun incident n'a été signalé ultérieurement, la patiente a passée 24 heures en réanimation sous une perfusion de sandostatine. Les valeurs du 5-HIAA urinaire étaient très élevées et l'examen histologique de la pièce opératoire a confirmé la nature carcinoïde de la tumeur. Nous soulignons la rareté de cette entité et l'intérêt de penser à une crise carcinoïde devons la survenue de complications peropératoires au cours d'une anesthésie pour tumeur de l'intestin grêle.

## Abstract (en)

Cardiac arrest in the operating room is a life-threatening event with multiple causes. We report the case of a 53-year old female patient with no particular past medical history scheduled for surgery to manage small intestine cancer. Twenty minutes after anesthetic induction the patient had asystole rapidly reversible after resuscitation measures. The association of face rash with chest rash gave rise to suspicion of late anaphylactic reaction. Rapid patient recovery allowed to resume surgical procedure. Tumor manipulation immediately caused a second severe bradycardia rapidly reversible after the administration of 0.5 mg atropine. Skin rush at the level of the face and the chest occurred again. This second complication immediately gave rise to suspicion of carcinoid crisis. Sandostatine was then administered. No other complication occurred, the patient spent 24 hours in the Intensive Care Unit receiving sandostatine infusion. Urinary 5-HIAA values were very high and histological examination of the surgical specimen confirmed carcinoid tumor. This study aims to highlight the rarity of this entity and the importance of suspecting carcinoid crisis in patients with intraoperative complications during anesthesia for small intestine tumor surgery.

## Introduction

L'arrêt cardiaque au bloc opératoire constitue un événement redoutable. Son incidence est estimée à 5,6 /10000 interventions [1], ces étiologies sont multiples. La crise carcinoïde est une des causes rares d'arrêt cardiaque peropératoire. A travers un cas clinique d'une asystolie rapidement réversible survenue au cours d'une anesthésie pour chirurgie d'une tumeur de l'intestin grêle et une revue de la littérature nous discutons la prise en charge de cette entité.

## Patient et observation

Il s'agissait d'une patiente de 53 ans, sans antécédents pathologiques particuliers notamment allergiques, hospitalisée pour une tumeur de l'intestin grêle. La symptomatologie clinique était de type digestif non spécifique: douleurs abdominales intermittentes, constipations.. L'imagerie par échographie et TDM abdominales, étaient en faveur d'un processus tumorale de l'intestin grêle associé à quelques adénopathies mésentériques sans notion de métastases décelables. L'indication d'une résection intestinale avec anastomose termino-terminale a était posée. L'évaluation pré-anesthésique était sans particularités: abdomen souple sans masse palpable, le bilan biologique était normal. Une prémédication à base d'hydroxyzine a était prescrite. Au bloc opératoire, la patiente est installée en décubitus dorsal avec mise en place d'un monitorage classique: cardioscopie, pression artérielle non invasive, saturation pulsé en oxygène. Deux voies veineuses périphériques de bon calibre ont été posées, remplissage au sérum physiologique 10 ml /kg, antibioprophylaxie par cefazoline 2g. L'induction est réalisée avec du propofol 3mg/kg, fentanyl 4ug/kg et cisatracurium 0,15 mg/kg. Une intubation orotrachéale a été effectuée facilement avec une sonde n°7,5 sans incidents avec courbe de capnographie (ETCO2) jugée satisfaisante, l'entretien anesthésique était à base d'oxygène-protoxyde d'azote (50:50) et isoflurane 2%. Environ 20 min après l'induction et au moment de l'incision chirurgicale, la patiente a présentée une bradycardie extrême (sans désaturation) suivie rapidement d'une asystolie avec chute de l'ETCO2, un rush cutané du visage et du thorax a été constatée. Un massage cardiaque externe a été rapidement effectué associé à une ventilation manuelle en oxygène pur, injection de 2 mg d'adrénaline avec un remplissage par 1000 ml de Ringer Lactate. Ces manœuvres ont permis rapidement en moins d'une minute la récupération d'une activité cardiaque en rythme régulier sinusal, d'une pression artérielle et d'un ETCO2 normales. Les pressions télé-expiratoires étaient normales et l'auscultation pulmonaire n'a pas retrouvé de sibilants, une réaction anaphylactoïde tardive a été suspecté motivant une injection de 120 mg de methylprednisolone. L'amélioration rapide de la patiente a permis l'autorisation d'une reprise de l'intervention chirurgicale. Le début de la manipulation chirurgicale de la tumeur a occasionné une deuxième bradycardie extrême jusqu'à 20 bat/min rapidement résolutive après 0,5 mg d'atropine et arrêt de la manipulation tumorale, une réapparition du rush cutané au niveau du visage et du thorax a été observée, cette deuxième complication a rapidement fait suspecter une crise carcinoïde conduisant à l'administration de 200 ug de sandostatine IV puis une perfusion continue de 100 ug/h, la poursuite de l'intervention a été autorisée une deuxième fois avec réalisation d'une résection et anastomose termino-terminale. Aucun incident n'a été signalé ultérieurement, la patiente a passée 24 heures en réanimation sous une perfusion de 100 ug/h de sandostatine, où un bilan dans le sens d'un syndrome carcinoïde a retrouvé des valeurs très élevées du 5-HIAA urinaire (5-hydroxy-indoleacetic acid): > 25 mg par 24 heures, l'examen histologique de la pièce opératoire a confirmé la nature carcinoïde de la tumeur.

## Discussion

L'arrêt cardiaque peropératoire peut survenir à n'importe quel moment et quelque soit la technique utilisée, les facteurs de risque rapportés sont l'anesthésie en urgence, les âges extrêmes et le score ASA élevé. Le pronostic est moins sombre qu'en extrahospitalier. Les étiologies les plus fréquemment rapportées sont: les hypoxies (difficultés d'intubation, ventilation inadéquate, bronchospasme..), les effets adverses des agents d'anesthésie ou adjuvants (surdosages, réactions anaphylactiques ou anaphylactoïdes, interférences médicamenteuses), et les causes cardiovasculaires (œdème pulmonaire, troubles du rythme ou de conduction, ischémie coronarienne, embolie) [2]. L'installation brutale de cette asystolie chez notre patiente et l'association du rash cutané nous ont orientés initialement vers l'étiologie anaphylactoïde, néanmoins le timing de cet incident loin de l'induction anesthésique (lors de l'incision chirurgicale), l'absence d'anomalies respiratoires et surtout la récidive lors de la manipulation tumorale sont des arguments qui ont permis de retenir l'étiologie carcinoïde plus que l'étiologie anaphylactoïde. Cette hypothèse à été confortée par l'absence de récidives après administration de sandostatine, puis confirmée par les taux élevés du 5-HIAA urinaire et la nature carcinoïde de la tumeur à l'étude histologique. Cet arrêt cardiaque serait le résultat de la libération de peptides vasoactifs dans la circulation systémique qui accompagne souvent les tumeurs carcinoïdes, on effet différents médiateurs peuvent être libérés au cours du syndrome carcinoïde (sérotonine, bradykinine, tachykinines, histamine, kallikreine, prostaglandines) [3], entrainant selon la nature de la molécule et ces interactions; une poussée hypertensive, hypotension, arythmie, bronchospasme et parfois même un arrêt circulatoire [4]. Le syndrome carcinoïde ne s'exprime qu'en présence de métastases hépatiques ou lorsque le drainage veineux de la tumeur échappe au système porte [5]. Au cours d'une intervention chirurgicale cette libération peut être déclenchée par l'induction, l'intubation, l'incision chirurgicale, la manipulation tumorale, et parfois sans facteur déclenchant évident [4]. Elle est aussi favorisée par l'utilisation d'agents sympathomimétiques ou histaminolibérateurs [6]. En préopératoire lorsque l'interrogatoire révèle l'existence d'un syndrome carcinoïde (bouffées vasomotrices, diarrhées, bronchospasme.), un dosage urinaire du 5-HIAA de 24 heures doit être effectué, un taux > 25 mg pose le diagnostic de tumeur carcinoïde [7,8], auquel cas une préparation par un analogue de la somatostatine est indiquée [7], elle permet souvent de supprimer ou du moins atténuer les manifestations de ce syndrome et de diminuer ainsi les risques périopératoires de survenue d'une crise carcinoïde [9]. Deux agents sont utilisés: l'octreotide (Sandostatine) et la lanreotide (SomatulineLP1). Ces agents freinent la libération des différents médiateurs et inhibent leurs effets au niveau des cellules cibles [9]. Lors d'une crise carcinoïde les manifestations sont en général résolutives à l'administration d'octreotide avec un délai d'action de quelques minutes [9]. Il est donc impératif de s'assurer de la disponibilité immédiate de cette molécule avant toute intervention chez un patient porteur d'une tumeur carcinoïde, même s'il est pris en charge pour un autre geste. Notre patiente n'a pas bénéficiée de cette préparation en raison de l'absence de signes cliniques pouvant orienter vers un syndrome carcinoïde, en plus le bilan d'extension n'a pas révélé l'existence de métastases hépatiques. Dans la littérature seulement 25% des tumeurs sécrètent activement des substances, et moins de 10% des personnes carcinoïdes peuvent développer le syndrome carcinoïde classique. Le foie étant capable d'inactiver rapidement ces substances vasoactives [10].

## Conclusion

L'évaluation préanesthésique de tout patient porteur d'une tumeur grêlique incite à rechercher activement un syndrome carcinoïde, sa présence indique formellement une préparation préopératoire par un analogue de la somatostatine. D'autre part la survenue de complications peropératoires au cours d'une anesthésie pour tumeur de l'intestin grêle doit faire penser à une crise carcinoïde.

## Conflits d’intérêts

Les auteurs ne déclarent aucun conflit d'intérêts.
